# Optimized 90° Pulse for Fast Measurement of Overhauser Magnetometer

**DOI:** 10.3390/s26082347

**Published:** 2026-04-10

**Authors:** Xiaorong Gong, Shuang Zhang, Shudong Chen, Xin Guo

**Affiliations:** 1 College of Electronic Science and Engineering, Jilin University, Changchun 130012, China; gongxr21@mails.jlu.edu.cn (X.G.); zhangshuang@jlu.edu.cn (S.Z.); 2 College of Physics, Jilin University, Changchun 130012, China

**Keywords:** Overhauser magnetometer, Larmor precession, magnetic field measurement, cycling rate

## Abstract

Overhauser magnetometer (OVM) is a proton precession magnetometer (PM) enhanced by electron resonance, and it is widely used in earthquake prediction, UXO detection, geological exploration, etc. For fast measurement, high cycling rate is necessary for OVM to enhance spatial resolution. Due to the impossibility to receive Larmor signal during the polarization process, traditional intermittent measurement is limited in fast mobile measurement applications owing to the long polarization time. Since it is difficult for proton magnetization to align rapidly for the long longitudinal relaxation time of liquid proton, we combined RF continuous excitation with a series 90° pulse to achieve fast measurement. To achieve the best alignment, a dynamic equation of Larmor precession is constructed and calculated, and the influences such as pulse waveform, pulse strength, and pulse duration on the proton magnetization alignment were investigated. The influence of different waveform pulses on the Larmor signal was studied experimentally, and the experimental results verified that the polarization time can be significantly shortened and fast measurement can be achieved by optimizing the waveform, strength, and duration of the 90° pulse. By using the optimized 90° pulse, the proton magnetization can be saturated within 3 ms, and 0.02 nT sensitivity was observed at 1 Hz cycling rate. Consistency between theory and the experiment indicates that the dynamic equation of Larmor motion can provide theoretical guidance for the investigation of fast measurement.

## 1. Introduction

Quantum magnetometers are precision instruments for weak magnetic field measurement [1,2]. Quantum magnetometers can be divided into proton magnetometer (PM) [3], Overhauser magnetometers (OVM) [4,5], optically pumped magnetometers (OPM) [6,7], and superconducting quantum interference devices (SQUID) [8] which have been widely used in mineral exploration [9], biology [10], geophysical exploration [11], satellite magnetic surveys [12], unexploded ordnance (UXO) detection [13], etc.

Cycling rate, the number of readings per second reported by the magnetometer, is an important technical indicator for any magnetometer. For mobile measurements such as airborne or marine magnetic surveys, the cycling rate determines the spatial resolution. Whether PM and OVM based on proton precession or OPM based on electron precession, polarization must be executed to force the orientation of the proton or electron magnetic moment perpendicular to the measured magnetic field. Only in this way can free precession be generated during the signal reception stage and frequency and magnetic field measurements be achieved. Therefore, the cycling rate is determined by both polarization time and reception time. The shorter the polarization and reception time, the higher the cycling rate, making it more suitable for fast measurement.

As instruments that calculate the magnetic field by measuring the Larmor precession frequency such as PM, OVM, and OPM, obtaining a free precession signal through polarization with a 90° pulse is a common method. Due to the long relaxation time of hydrogen protons in liquids, polarization fields generated by strong DC pulses to achieve magnetic moment alignment require long polarization time (about 2 s). The cycling rate is usually only 1/3 causing difficulty for PM to meet the demand for high cycling rates [14]. Moreover, this polarization method has drawbacks such as high power consumption due to large polarization currents, and low signal-to-noise ratio (SNR) compared to OVM and OPM [15] owing to low polarization efficiency [16]; OPM employs the optical pumping to excite electromagnetic magnetic resonance for magnetic measurement [17]. Due to the short relaxation time of electrons, polarization saturation can be achieved with only several milliseconds [12]. Moreover, the high gyromagnetic ratio of electrons is beneficial for shortening the reception time to obtain high cycling rates and sensitivity [12]. All of these make OPM particularly suitable for fast measurement. Unlike PM and OPM, OVM employs dynamic nuclear polarization (DNP) to enhance proton polarization [4,5], exhibiting no heading errors and blind zones and achieving sensitivity levels comparable to cesium-based OPMs, and this makes OVM and OPM have the same application scenarios, especially in low-power applications [18].

Because of the fact that OVM is a proton precession magnetometer enhanced by electrons, its polarization process is more complex than PM and OPM. So far, there are few reports on theoretical and experimental research dedicated to fast measurement from a microscopic perspective. At present, most reports use long pulse polarization methods similar to PM, which typically have a cycling rate of only 1/3 [19]. Wang et al. [20] introduced a fast OVM measurement technique for marine surveys using continuous RF excitation combined with short transverse bias fields. However, the reported sensitivity at 1 Hz was only 0.05 nT. Lower sensitivity means poor SNR of the Larmor signal, and it restricts the application of OVM for rapid measurement. Similar to the literature [20], continuous RF excitation combined with short transverse bias fields is used in this paper, the difference is that we studied the influence of different polarization pulses on the Larmor signal. These studies reveal relationship between the polarization pulses and proton magnetic moment alignment, Larmor signal and sensitivity.

The main contributions of this work are as follows:

(1) In Section 3, we derive and analyze the dynamic equation governing Larmor precession under 90° pulse excitation, enabling theoretical prediction of optimal pulse parameters.
(2) In Section 4, based on the analysis, we design and implement a high cycling rate OVM using optimized pulse waveforms to achieve efficient polarization.
(3) In Section 5, we experimentally validate the proposed method under both natural and artificial magnetic field conditions, demonstrating improved sensitivity and cycling rate.

## 2. The Working Principle of OVM

Compared with traditional PM with strong direct current (DC) polarization (about 1 A), OVM uses the DNP effect to enhance the polarization of protons. DNP effect takes advantage of the strong dipole–dipole coupling between unpaired electrons and protons to enhance the nuclear magnetic resonance (NMR) signal through polarization transfer from electron spin to nuclear spin via cross-relaxation mechanisms [21].

**Figure 1:**
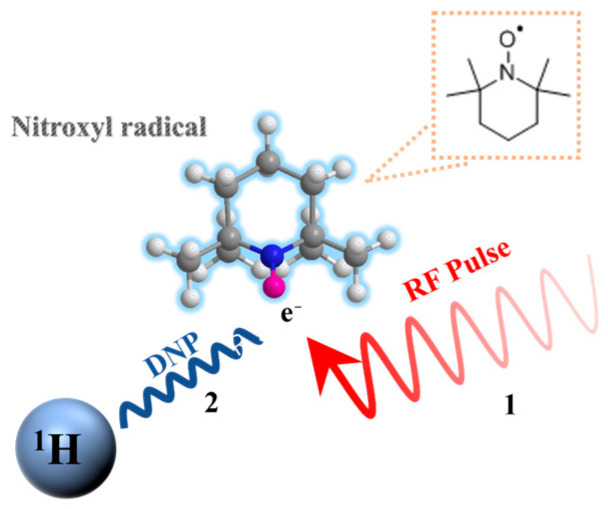
Dynamic nuclear polarization process.

The DNP in liquids can be divided into two phases (see Figure 1). Firstly, the RF pulse excites the unpaired electrons in free radicals to generate electron spin resonance (ESR) [22]. Secondly, this non-equilibrium population is then transferred to nuclear spin from electron spin due to molecular diffusion yielding an enhancement of the NMR signal of solvent protons [23]. The enhancement of the nuclear, upon irradiation of the ESR, is called the dynamic nuclear polarization factor (DNPF). The DNPF of protons in the Earth’s magnetic field can reach around 2000 [24], greatly improving the signal and sensitivity of the instrument.

**Figure 2:**
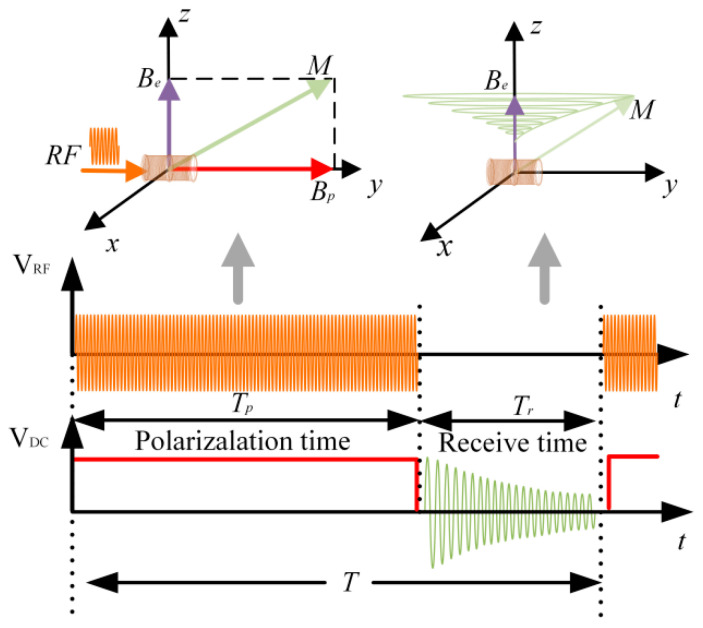
Timing diagram intermittent measurement method.

In the Earth’s magnetic field, the excitation of a free radical solution with RF pulses to generate DNP results in an enhanced proton macroscopic spin magnetization ***M***. Because ***M*** is paralleled to the Earth’s magnetic field ***B***_e_, it is unable to precess around the Earth’s magnetic field to generate Larmor signals. In order to generate Larmor signals, a common method is to simultaneously use RF polarization and DC polarization to enhance the proton magnetization in the direction of the total magnetic field of the bias field and the Earth’s magnetic field (see Figure 2). After the proton macroscopic spin magnetization ***M*** is saturated, both RF and DC polarization are turned off simultaneously. Then, the magnetization ***M*** will precess around the Earth’s magnetic field, which is called Larmor precession.

During Larmor precession, the induction signal is generated by the magnetization ***M*** in the coil, which is called the free induction decay (FID) signal. The OVM measures the frequency of the Earth’s magnetic field by calculating the frequency of the FID signal. The relationship between the angular frequency *ω* of the FID signal and the proton gyromagnetic ratio $\gamma_{p}$ is

(1) $$\omega =\gamma_{p}B.$$

where the value of $\gamma_{p}$ is 2.6752118744 × 10^8^ s^−1^T^−1^, which is recommended by CODATA in 2018.

According to (1), due to the presence of a static polarization field during the polarization process, the precession frequency of the Larmor signal is not the frequency of the Earth’s magnetic field itself. Therefore, a challenge of the intermittent measurement method is that polarization and acquisition cannot be performed simultaneously. Due to the long longitudinal relaxation time *T*_1_ of protons in liquids, a larger FID signal amplitude requires a longer polarization time. Therefore, the cycling rate of the intermittent measurement method is usually greater than 3 s, which makes this method difficult to use for fast measurement.

**Figure 3:**
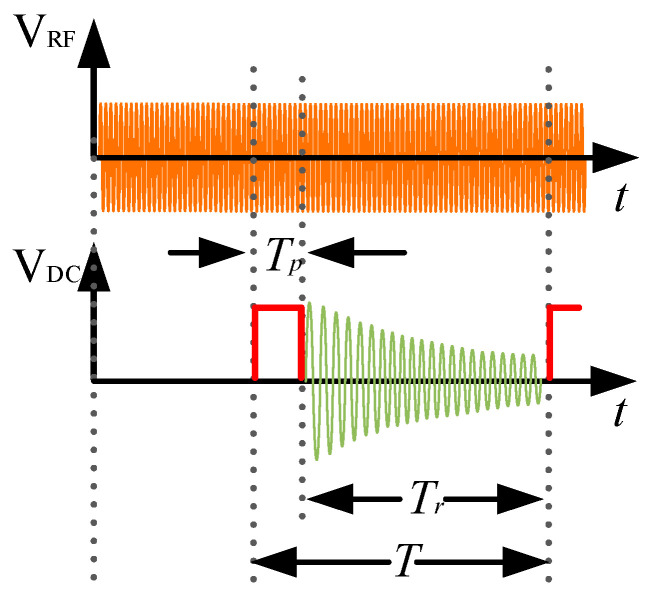
Timing diagram of 90° pulse method.

As mentioned above, the length of the polarization time determines the cycling rate. To achieve fast measurement, the polarization time must be greatly reduced. Because the RF frequency is tens of MHz and the Earth’s magnetic field is 20,000~70,000 nT (corresponding to 850~3000 Hz), the RF polarization will not affect the signal acquisition process and it is always in the on state during the whole measurement process. A very short DC pulse before signal acquisition at every cycle to generate the transverse magnetization component, as shown in Figure 3, which is called 90° pulse method for fast measurement.

Usually, the longitudinal relaxation process of hydrogen protons in PM takes 2 s. OVM has a higher polarization efficiency using the DNP technique, which allows us to attempt to reduce the polarization time by using short polarization pulses. But what is the relationship between pulse waveform, pulse width, pulse strength and Larmor signal? The answers to these questions will guide us in selecting the optimal pulse for fast measurement. Following, we will seek answers to these problems through theoretical analysis of the dynamic equation of proton magnetization.

## 3. Theory for Fast Measurement

### 3.1. Equations Construction of the Proton Magnetization Dynamics Equation

**Figure 4:**
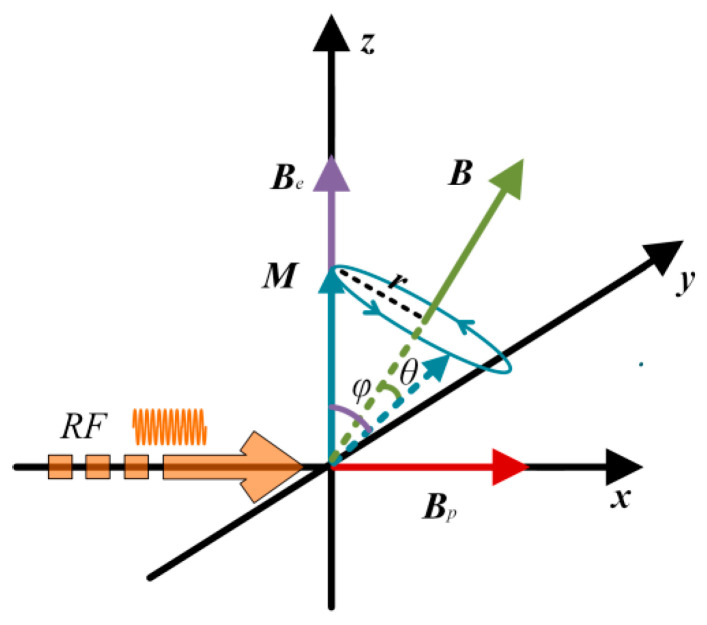
Schematic diagram of the movement of the endpoint of the magnetization ***M*** vector.

Under the excitation of the RF, the magnetization ***M*** is enhanced along the Earth’s magnetic field ***B***_e_ as shown in Figure 4. When a 90° DC pulse is applied, the polarization field ***B***_p_ and the Earth’s magnetic field ***B***_e_ form a total magnetic field ***B***. At this time, the magnetization ***M*** will be subjected to a magnetic torque perpendicular to the plane formed by ***M*** and ***B***. Under the action of this torque, the moment ***M*** will precess around the total magnetic field ***B***. The motion trajectory of the endpoint of the vector ***M*** is a circle. If the angle between ***M*** and ***B*** is *θ*, and the radius of the circle is *r*, we have

(2) $$r=\left|M\right|\sin\theta .$$

The relationship between ***M*** and angular frequency *ω* can be expressed as:

(3) $$v=\left|\frac{dM}{dt}\right|=r\omega .$$

According to (3), the vector equation of (3) can be written as follows:

(4) $$\frac{dM}{dt}=\left|\frac{dM}{dt}\right|\frac{B\times M}{\left|B\times M\right|}=r\omega \frac{B\times M}{BM\sin\theta }.$$

Combined (1), (2) and (4), we have

(5) $$\frac{dM}{dt}=\gamma_{p}B\times M.$$

From (5), we can see that this equation is the Bloch equation [1], which is a dynamic equation describing the proton magnetization.

### 3.2. Equation Solution of Magnetization Dynamic Equations

For our purposes, we are interested in the influence of ***B***_p_ on the trajectory of the magnetization ***M*** endpoint and analyze the trajectory of the magnetization ***M***. If the time scale in (5) is very small, we can use the differential element method to approximate (5), and we have

(6) $$\frac{dM(t)}{dt}=\frac{M\left(t+\Delta t\right)-M\left(t-\Delta t\right)}{2\Delta t}.$$

Substituting (6) in (5), we obtain

(7) $$M\left(t+\Delta t\right)=M\left(t-\Delta t\right)+2\Delta t\gamma_{p}B\left(t\right)\times M\left(t\right).$$

Replace time variable (t+∆t) with *t*, we have

(8) $$M\left(t\right)=M\left(t-2\Delta t\right)+2\Delta t\gamma_{p}B\left(t-\Delta t\right)\times M\left(t-\Delta t\right).$$

According to (8), if the time scale ∆t is set small enough, the value of the moment ***M***(*t*) is determined by the ***M***(*t* − Δ*t*), ***M***(*t* − 2Δ*t*) and ***B***(*t* − Δ*t*). Now, we can use (8) to discuss the motion law of the magnetization ***M***.

## 4. Calculation and Discussion

In order to improve the polarization efficiency of the 90° pulse, this paper proposes a gradual 90° pulse DC polarization method, and the timing diagram is similar to Figure 3. The only difference is that the 90° DC pulse increases gradually. In this process, the total magnetic field ***B*** of the DC polarization field ***B***_p_ and the Earth’s magnetic field ***B***_e_ slowly deflect from the *z*-axis to the *y*-axis. At this time, under the action of the total magnetic field ***B***, the proton magnetization is slowly deflected from the *z*-axis to the *y*-axis, and precesses around the total magnetic field ***B*** with an extremely small radius, ultimately achieving a 90° deflection of the proton magnetization.

The polarization field ***B***_p_ can be expressed as

(9) $$B_{p}(t)=B_{0}{\left(t/t_{p}\right)}^{\alpha },$$

where *B*_0_ is the pulse strength, *t*_p_ is the pulse duration, and *α* is the pulse waveform index. Different index *α* correspond to different pulse waveforms. From (9), we can see that the polarization field ***B***_p_(t) is affected by three factors: the waveform, strength, and duration of the pulse. The influence of each factor on the polarization effect will be discussed below.

In order to estimate the deflection efficiency of the 90° pulse, we define the polarization saturation factor *θ* to represent the angle between the magnetization ***M*** and the total magnetic field ***B*** after the polarization is completed. The smaller the saturation factor *θ*, the more effectively the proton magnetization ***M*** is deflected to the total magnetic field ***B*** direction.

### 4.1. The Influence of 90° Pulse Waveform on Polarization Efficiency

**Figure 5:**
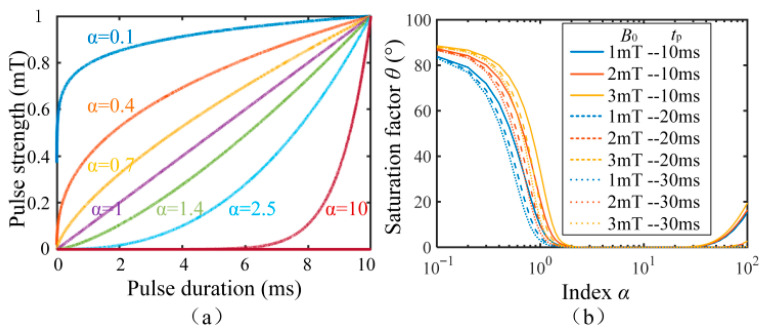
(**a**) Pulse waveform vs. index *α*; (**b**) saturation factor *θ* vs. index *α*.

According to (9), when the pulse strength is 1 mT and the pulse duration is 10 ms, the pulse waveforms under different α values are shown in Figure 5a. Figure 5a indicates that changing the pulse waveform index *α* affects the shape of the pulse waveform. To investigate the influence of different shapes of the pulse waveforms on the polarization saturation factor *θ*, we suppose that the modulus of the initial magnetization vector strength ***M***(0) is 10^−7^ A/m [25], and the direction of the initial magnetization vector is aligned with the Earth’s magnetic field, where the Earth’s magnetic field is 54 uT. The effect of index *α* on the polarization saturation factor *θ* at different pulse strengths and pulse duration is calculated according to (8). The calculated results are shown in Figure 5b.

Figure 5b shows that the smaller the index *α* is, the larger the polarization saturation factor *θ* is. This means that the rapid enhancement of pulse strength in the early time is not beneficial for achieving high polarization efficiency. The saturation factor *θ* is close to zero when *α* is greater than 2, and this indicates that the proton magnetization vector has been completely deflected to the direction of the total magnetic field ***B***. We also see that the saturation factor *θ* is enhanced again when α is larger than 30, and this indicates that enhancement of pulse strength, whether too fast or too slow in the early time is not beneficial for achieving high polarization efficiency.

**Figure 6:**
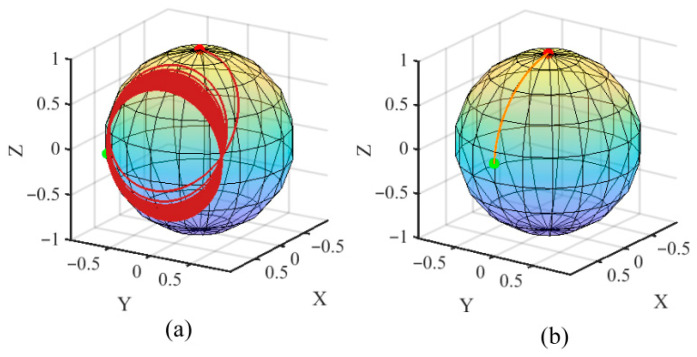
The motion trajectory of the endpoint of the proton magnetization vector under different *α.* (**a**) Index *α* = 0.5; (**b**) Index *α* = 10.

**Figure 7:**
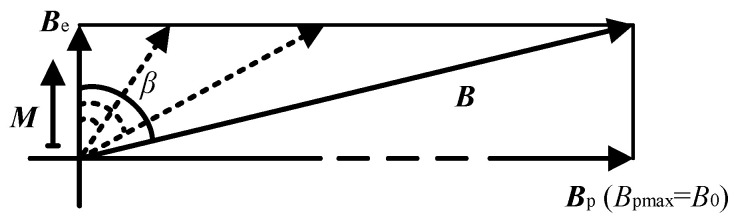
Angle *β* of total field ***B*** change with polarization field ***B***_p_.

To explain the above phenomenon, the motion trajectory of the endpoint of the proton magnetization vector at different index *α* was simulated when the pulse strength was 3 mT and the pulse duration was 20 ms. The results are shown in Figure 6. The red point and the green point in Figure 6 represent the start position and endpoint of the motion trajectory of the endpoint of the ***M***, respectively. In the case of Figure 6a, from ***B***_p_(t) = 0 to ***B***_p_(t) = *B*_pmax_, the change in the direction *β* of the total field ***B*** occurs with rapidly increasing speed as shown in Figure 7.

At this time, the angular velocity of the proton magnetization ***M*** cannot keep up with the speed of change in the total field ***B***, which results in the final magnetization spiraling around the total magnetic field with a larger radius (the polarization saturation factor *θ* = 44.7° at last), and cannot be aligned to the direction of the total magnetic field. In the case of Figure 6b, as shown in Figure 7, from ***B***_p_(t) = 0 to ***B***_p_(t) = *B*_pmax_ to the change in the direction *β* of the total field ***B*** occurs with a moderate increasing speed. In this time, the angular velocity of the proton magnetization is close to the speed of change in the total field ***B***, so that in the whole process, the magnetization can spiral around the total magnetic field with a very small radius and effectively aligned with the direction of the total magnetic field (the polarization saturation factor *θ* = 0.005° at last).

Therefore, whether the early total field changes rapidly (small *α*) or the late total field changes rapidly (large *α*), it will lead to a decrease in polarization efficiency. As clearly shown in Figure 5b, the polarization saturation factor *θ* approaches zero when the waveform index α is between 2 and 30. This optimal range signifies that the proton magnetization vector ***M*** can follow the direction of the total magnetic field ***B*** with a minimal precession radius throughout the pulse duration. Consequently, only with an appropriate α within this range can ***M*** be effectively aligned nearly perpendicular to the Earth’s magnetic field upon the removal of ***B***_p_, thereby maximizing the transverse magnetization component for signal acquisition.

### 4.2. The Impact of 90°Pulse Strength on Polarization Efficiency

**Figure 8:**
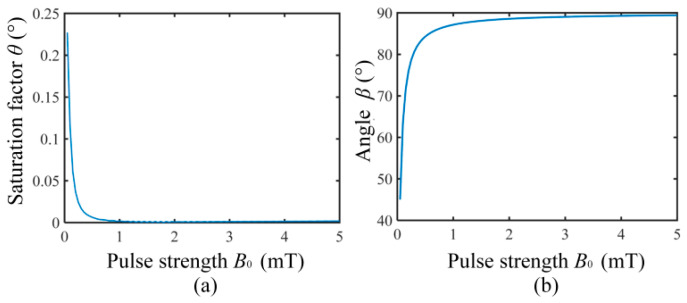
(**a**) Pulse strength *B*_0_ vs. polarization saturation factor *θ*; (**b**) the angle *β* vs. pulse strength *B*_0_.

In order to analyze the relationship between the pulse strength and the polarization saturation factor *θ*, simulations were conducted based on (8) and (9) under the following conditions: the pulse index *α* was set to 3, the pulse duration to 20 ms, the Earth’s magnetic field to 54 µT, and the initial magnetization strength ***M***(0) to 10^−7^ A/m. The pulse strength *B***_0_** was incremented from 0.05 mT to 5 mT in steps of 0.05 mT. The simulation results shown in Figure 8a indicate that the influence of pulse strength *B***_0_** on polarization saturation factor *θ* is negligible when *B***_0_** is several mT. That is to say, polarization orientation can be achieved in several tens of milliseconds with several mT pulse amplitudes.

However, from the effect of the pulse strength on *β* in Figure 7, it can be seen that the final deflection position of magnetization ***M*** depends on the direction of the total magnetic field ***B*** of the polarization pulse strength *B*_0_ and the Earth’s magnetic field ***B***_e_. The angle β between the total magnetic field ***B*** and the earth’s magnetic field can be expressed as

(10) $$\beta =\mathrm{arc}(\frac{B_{e}\cdot B}{\left|B_{e}\right|\left|B\right|}).$$

According to (10), we calculated the angle *β* between the total magnetic field ***B*** and the Earth’s magnetic field when the pulse strength *B*_0_ ranges from 0 to 5 mT, and the results are shown in Figure 8b. Figure 8b shows that the greater the strength *B*_0_ of the polarization pulse, the greater the angle *β* of the total magnetic field ***B*** away from the Earth’s magnetic field. When the pulse strength *B*_0_ is greater than 3 mT, the angle *β* almost reaches 90°, with the same direction to the total magnetic field parallel to the *y*-axis. Compared with the traditional PM method, in which the polarization current is ampere-level and the polarization field is tens of mT, the polarization current and the polarization field are tens of mA and several mT, respectively.

### 4.3. The Impact of 90°Pulse Duration on Polarization Efficiency

**Figure 9:**
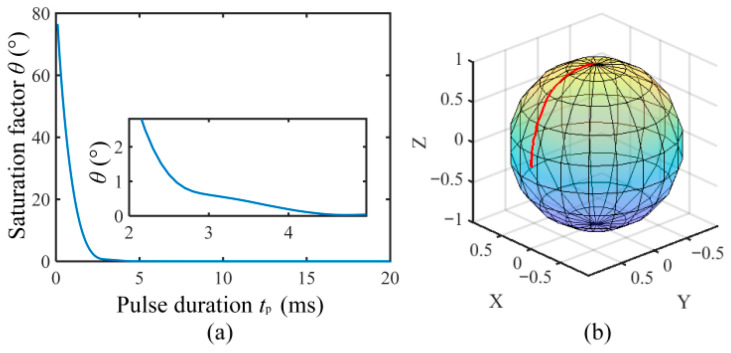
(**a**) Pulse duration vs. saturation factor *θ*; (**b**) the trajectory of 90° pulse magnetization ***M*** at *α* = 3, *B*_0_ = 3 mT and *t*_p_ = 3 ms.

In the above simulations, a relatively long polarization time is selected for the 90° pulse. However, the longer the polarization time, the shorter the acquisition time of the FID signal, this is not beneficial to high sensitivity. Therefore, it is necessary to analyze the impact of pulse duration *t*_p_ on the polarization saturation factor. Considering (8) and (9), we assume that the pulse index is set to 3, the pulse strength is 3 mT, the Earth’s magnetic field is 54 µT, and the initial magnetization strength ***M***(0) is 10^−7^ A/m. The pulse duration is simulated from 0.1 ms to 20 ms, with a step size of 0.1 ms. The polarization saturation factor corresponding to different pulse durations is shown in Figure 9a.

Figure 9a shows that when the pulse duration is less than 2 ms, the saturation factor decreases rapidly with the increase in the pulse duration; on the contrary, when the pulse duration is greater than 3 ms, the saturation factor *θ* is less than 1. This phenomenon can be explained by the abrupt polarization field causes a strong change in the total magnetic field. This causes that the angular velocity of the proton magnetization cannot keep up with the speed of change in the total field ***B***, which results in the final proton magnetization ***M*** spiraling around the total magnetic field with a larger radius and polarization saturation factor *θ*, and this is similar to the situation described in Figure 6a. So we concluded that the shortest pulse duration is 3 ms.

Through the above simulation analysis, we can obtain the range of the optimal waveform index *α*, strength *B*_0_, and duration time *t*_p_ of the 90° pulse which is listed in Table 1. According to the recommended parameters given in Table 1, we simulated the endpoint motion trajectory of the magnetization ***M***. The simulated results shown in Figure 9b indicate that the magnetization ***M*** can be effectively deflected from the *z*-axis to the *y*-axis in milliseconds.

## 5. Experiment

### 5.1. Magnetometer Used for Experiments

**Figure 10:**
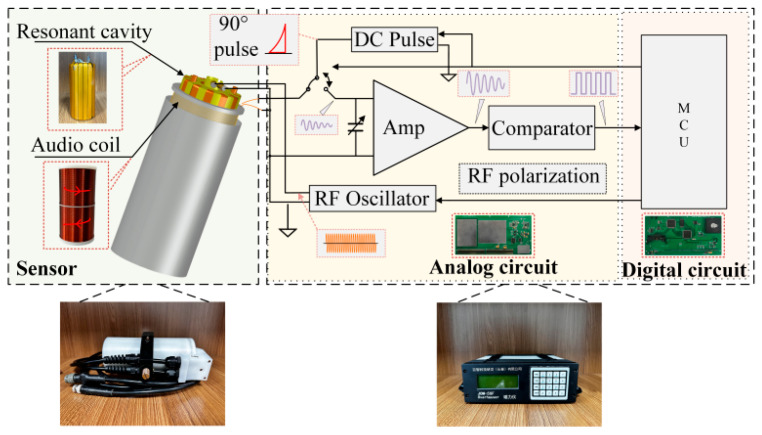
JOM-5SF OVM system diagram.

As is shown in Figure 10, the prototype JOM-5SF OVM is developed by the College of Electronic Science and Engineering, Jilin University [19]. The OVM sensor consists of a resonant cavity, an audio coil, and a quartz bottle filled with a free radical solution. The free radical solution is ^15^N-D-4-Oxo-2,2,6,6-tetramethylpiperidine-1-oxol (^15^N-D-oxo-TEMPO). The quality factor of the resonant cavity is approximately 1500, the resistance of the audio coil is 21.6 Ω, and its inductance is 33.5 mH. When RF energy is injected into the resonant cavity, a circular RF polarization field is generated in the cavity to excite the ESR, thereby enhancing the macroscopic magnetization of the proton. The audio coil is used both to generate a 90° pulse polarization field and receive the Larmor precession signal. A reverse series winding structure is used to enhance the anti-interference ability of the audio coil.

The console of the OVM consists of an analog board and a digital board. In the polarization stage, after receiving the signal sent by the microcontroller unit (MCU), the analog circuit generates the 90° pulse bias signal and the RF polarization signal. During the acquisition process, the FID signal is resonantly amplified in the range of 20 μT to 120 μT through the LC resonant circuit. The tuned FID signal is amplified to the volt level by the amplifier circuit, and a square wave signal is output after passing through the hysteresis voltage comparator circuit. It is transmitted to the MCU for measuring frequency, and the relationship between the magnetic field and the measured frequency value is calculated by (1).

### 5.2. Experimental Results of Different 90° Pulse Parameters

To verify the effectiveness of the optimized 90° pulse polarization method proposed in this study, we designed a series of experiments to analyze the influence of pulse waveform index *α*, pulse strength *B*_0_, and pulse duration *t_p_* on the Larmor signal.

The experimental control system for generating optimized 90° pulses is implemented through a specific path combining high-precision digital pulse width modulation (PWM) with digital-to-analog conversion (DAC): First, a baseband PWM signal with a fixed carrier frequency (e.g., 25 kHz) is generated by a timer within the microcontroller unit (MCU), and the required analog waveform shape is defined by programming the duty cycle of this PWM signal in real time and with precision. This shape is determined by Formula (9), and the MCU updates the duty cycle value in each control cycle based on the calculated expected voltage trajectory. Subsequently, the digital PWM signal is fed into a high-linearity PWM-to-voltage conversion circuit (using the LTC2644 DAC chip), which acts as a 1-bit digital-to-analog converter, performing low-pass filtering and buffering on the PWM signal to output a smooth and precise analog voltage proportional to the instantaneous PWM duty cycle. Then, this analog voltage is amplified by a power amplifier and drives the audio coil in the sensor, generating a time-varying current in the coil that strictly follows the programmed voltage waveform, thereby generating the required time-varying magnetic field pulse ***B***_p_(t) within the sensor’s sensitive volume. By using different duty cycle–time relationships (i.e., different exponent α values) in the preset software, the system can flexibly generate various optimized pulse waveforms as shown in Figure 5a.

All experiments were conducted in a natural geomagnetic environment. The Larmor signal was acquired using a handheld oscilloscope (Rohde & Schwarz RTH1002). During the experiment, the parameters of the 90° pulse were selected using the recommended values in Table 1. In order to observe the effects of different pulse parameters, two of the recommended parameter values were fixed in the experiment, and the single parameter to be observed was changed for testing. This can effectively analyze the specific effects of each parameter on the experimental results and ensure that the effects of the adjusted parameters are accurately evaluated when other conditions remain unchanged.

**Figure 11:**
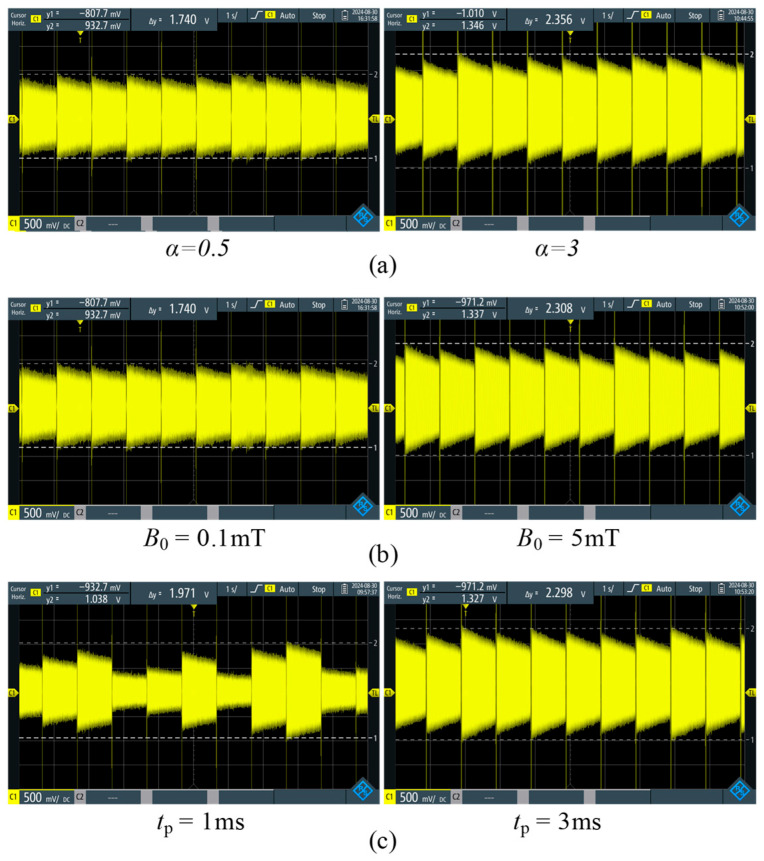
Measurement results of Larmor signal under different pulse waveforms. (**a**) pulse index α; (**b**) pulse strength *B*_0_; (**c**) pulse duration *t*_p_.

The influence of different polarization waveforms on the Larmor signal is shown in Figure 11a. When the waveform index *α* = 0.5, the amplitude of the Larmor signal is small compared to *α* = 3. The reason is that the precession radius of the proton magnetization vector around the total magnetic field is too large, and it is consistent with the theoretical simulation that the saturation factor is 81° when the exponent *α* = 0.5. As shown in Figure 11a, the amplitude of the Larmor signal is unstable, with a maximum peak-to-peak value from 1 V to 1.8 V. From Figure 4, we can see that the relative position of the magnetization and the total magnetic field is random when each pulse polarization is completed, resulting in a random angle *φ* between the magnetization vector and the Earth’s magnetic field. Since the amplitude of the signal is proportional to the transverse component of the magnetization ***M*** [20], and the effective value of the transverse component ***M***_y_ can be represented by (11), the random variation in the angle *φ* is the reason for the instability of the signal. When index *α* = 3, the amplitude of the Larmor signal is stable, with a peak-to-peak value of 2.3 V. This result shows that the precession radius of the magnetization around the total magnetic field is small, with a polarization saturation factor is 0.7°. At this time, the proton magnetization is completely aligned with the direction of the total magnetic field.

(11) $$M_{y}=M\sin\varphi .$$

The influence of different pulse strengths on the Larmor signal is shown in Figure 11b. When *B*_0_ = 5 mT, the total magnetic field is almost parallel to the *y*-axis, and the amplitude of the Larmor signal is the same as in Figure 11a (*B*_0_ is 3 mT). However, when *B*_0_ = 0.1 mT, the Larmor signal amplitude is reduced by 0.57 V compared with *B*_0_ = 5 mT. This shows that when the pulse strength is too small, the total magnetic field has a large angle with the *y*-axis, and the magnetization is not completely deflected to the *y*-axis. From the comparison between Figure 11a,b, we see that the influence of pulse strength on *θ* is not significant, which is consistent with the theoretical simulation.

The effect of different pulse durations on the Larmor signal is shown in Figure 11c. When the pulse duration *t*_p_ is 3 ms, the Larmor signal is relatively stable. This indicates that the radius of magnetization around the total magnetic field is small, which is consistent with the theoretical *θ* = 0.8°. On the contrary, when *t*_p_ = 1 ms, the Larmor signal amplitude is both small and unstable. It is due to proton magnetization not being able to keep up with the speed of change in the total field ***B***, resulting in an excessively large precession radius and it is consistent with the theoretical *θ* = 27°. From the comparison between Figure 11a (*θ* = 81°) and Figure 11c (*θ* = 27°), we see that the Larmor signal corresponding to *θ* = 81° is even worse than corresponding to *θ* = 27°. Similar to the factor *α* affecting signal stability, the random variation in the angle *φ* is the reason for the instability of the signal. The smaller the *θ*, the more stable the *φ* and the signal amplitude angle *φ* is the reason for the instability of the signal. The smaller the *θ*, the more stable the *φ* and the signal amplitude.

**Figure 12:**
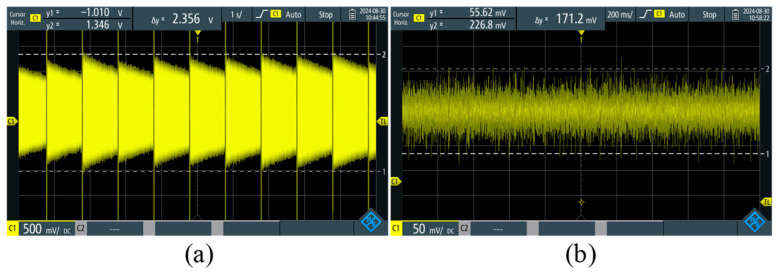
(**a**) FID signals after optimization; (**b**) JOM-5SF background noise.

Then, we measured according to the pulse parameters recommended in Table 1, and its Larmor signal is shown in Figure 12a, with an amplitude of 1.15 V. In addition, the FID signal noise, as shown in Figure 12b, is 26 mV according to (12).

(12) $$s=\sqrt{\frac{1}{n-1}\sum_{i=1}^{n}{\left(x_{i}-\bar{x}\right)}^{2}},$$

where $x_{i}$ is the value of *i* sampling points, $\bar{x}$ is the average of all sampling points. After calculation, the initial maximum SNR of the FID signal is 44.

### 5.3. Sensitivity Estimation in Natural Environment

**Figure 13:**
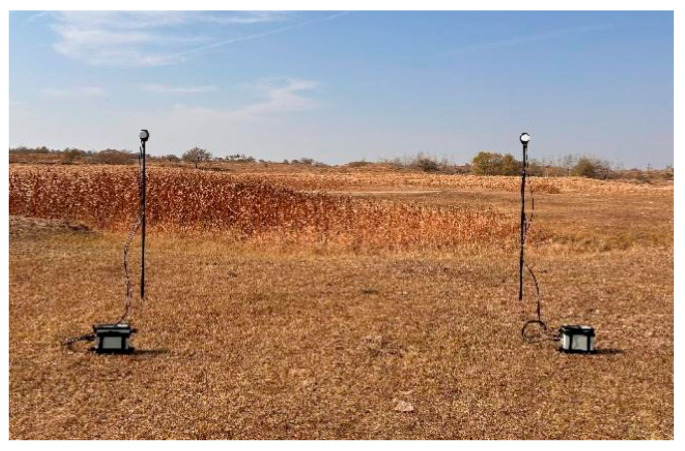
Two synchronized JOM-5SF in natural environment.

In the natural environment, two synchronous instruments are used to evaluate the performance of the 90° pulse proposed in this paper. A rural area far away from the urban area was selected to reduce electromagnetic interference. The two synchronous instruments are separated by 5 m to avoid mutual interference; the sensors are supported by metal rods to avoid the influence of ground current and magnetic materials. The cycling rate is set to 1 s, and the field test setup is shown in Figure 13.

**Figure 14:**
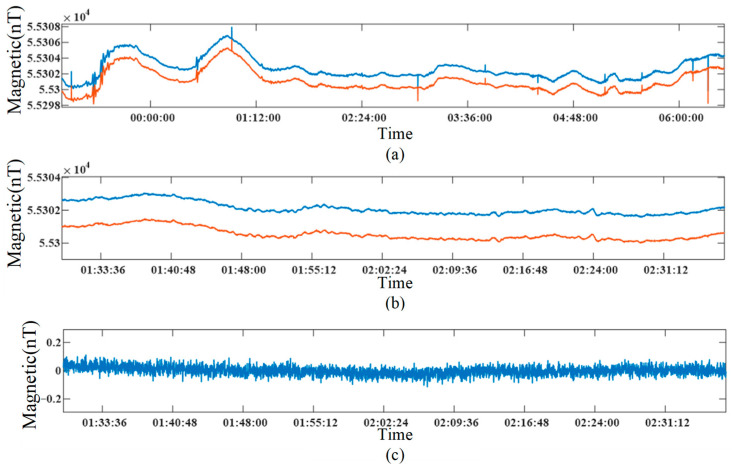
(**a**) Measured magnetic field of two synchronized JOM-5SF; (**b**) magnetic field measured from 1.30 am to 2.30 am; (**c**) difference results of two JOM-5SF. Blue: Measurement results of the first JOM-5SF prototype. Orange: Measurement results of the second JOM-SF prototype.

To reduce the human magnetic disturbance on the measurement, two synchronized JOM-5SF magnetometers were used to measure the Earth’s magnetic field between 11:00 pm and 7:00 am the next day, as shown in Figure 14a. Due to the gradient of the Earth’s magnetic field, the two measurement curves do not overlap, but the trends of the magnetic field fluctuations and the spark interference are highly consistent. The noise of the magnetometer is characterized by the relative uncertainty of the repeated measurement of the magnetic field value, also known as sensitivity. Usually, the standard deviation (STD) of the magnetic field data is used to describe the sensitivity of the magnetometer quantitatively.

In the natural environment, due to the diurnal variation in the Earth’s magnetic field and electromagnetic interference, the sensitivity is difficult to evaluate with a single instrument. However, the influence of diurnal variation and electromagnetic interference can be effectively eliminated by subtracting the data of the two synchronous instruments. In addition, the stable magnetic field measured from 1.30 to 2.30 in the morning, as shown in Figure 14b, with low electromagnetic interference (EMI) was selected to evaluate the sensitivity of the instrument more accurately. The difference results of the magnetic field values of the two instruments are shown in Figure 14c. The results show that the difference effectively removes the low-frequency diurnal variation and EMI. Finally, the sensitivity is 0.0217 nT, which is calculated by (13).

(13) $$\sigma =\frac{1}{\sqrt{2}}\sqrt{\frac{1}{n-1}\sum_{i=1}^{n}{\left(B_{1i}-B_{2i}-{\bar{B}}_{1i}+{\bar{B}}_{1i}\right)}^{2}},$$

where *B*_1*i*_ and *B*_2*i*_ are the magnetic field values of the two instruments, and *B*_1*i*_ and *B*_2*i*_ are the average values of *B*_1*i*_ and *B*_2*i*_, respectively.

### 5.4. Sensitivity Estimation in Artificial Magnetic Field System

**Figure 15:**
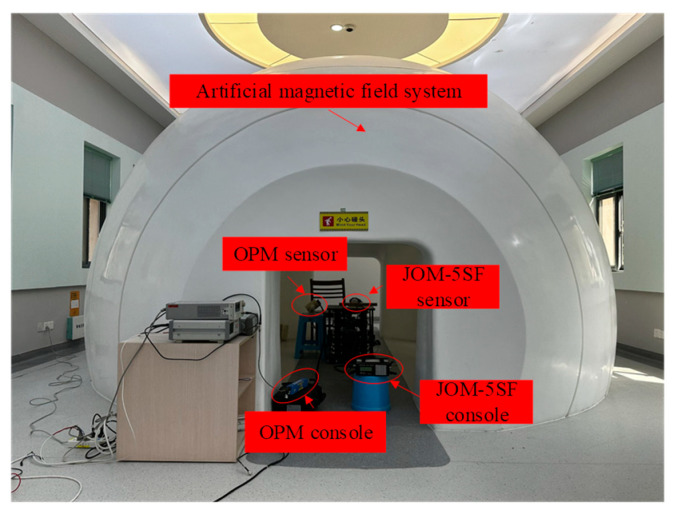
Artificial magnetic field measurement setting.

In the natural magnetic field environment, there are many unpredictable factors, such as diurnal variation and EMI, so the above evaluation results can only be used as a reference for instrument sensitivity. To accurately evaluate the sensitivity of the proposed 90° pulse, we conducted experiments at the “First Class Weak Magnetic Metering Station of NDM” of the 710th Institute (Yichang, China). The experiment setup is shown in Figure 15.

The artificial magnetic field system consists of a magnetic shielding room, a Helmholtz coil system for generating uniform magnetic fields, and a compensated OPM (CAM-01, 710th Institute, Yichang, China) serving as the reference sensor. The CAM-01 outputs a frequency signal proportional to the magnetic field strength, which is phase-compared with a reference frequency from a signal generator. The phase difference is converted by a phase detector into a voltage that drives the compensation coil, thereby generating a stable magnetic field. The peak-to-peak noise level of the CAM-01 is 0.002 nT, and the background noise of the uniform magnetic field system is approximately 0.005 nT, both significantly lower than the sensitivity level of the developed system. In addition, the uncertainty of the artificial magnetic field is 0.02 nT, which can be used to calibrate the absolute accuracy of the OVM.

**Figure 16:**
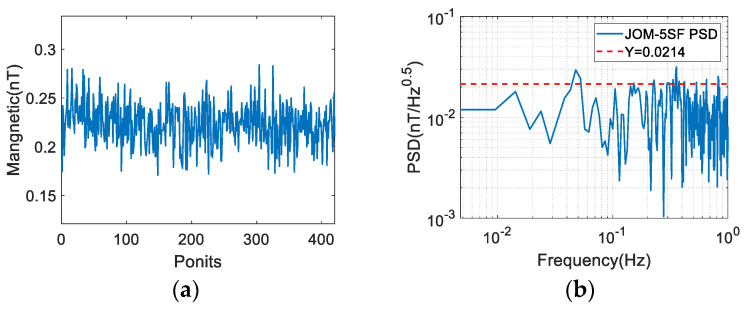
(**a**) Time domain of artificial field magnetic field test results; (**b**) PSD analysis of magnetic field measurement results.

The results of fast measurement using this 90° pulse method are shown in Figure 16a. The overall fluctuation of the measured magnetic field is less than 0.1 nT. Since there is no low-frequency interference such as diurnal variation, the sensitivity of a single instrument can be directly expressed by the STD, and the measured result is 0.021 nT, which is comparable to the sensitivity in the natural environment. This shows that the method of evaluating sensitivity using two synchronous measuring instruments in a field environment is reliable.

In addition, the sensitivity of the magnetometer can also be evaluated by power spectral density (PSD), and the results of the reading noise spectrum analysis of the magnetic field value are shown in Figure 16b. The instrument’s sensitivity was confirmed to order 0.021 nT/$\sqrt{Hz}$, comparable to direct STD result in the time domain STD result.

Table 2 shows the absolute error between the JOM-5SF OVM and the standard field under different magnetic field strengths (20–80 μT) generated by an artificial magnetic field. The results indicate that within this field strength range, the absolute error of the JOM-5SF remains within ±0.1 nT.

## 6. Conclusions

The traditional intermittent measurement method achieves polarization saturation of the proton magnetization by extending the polarization time so as to obtain a considerable Larmor signal amplitude. In order to reduce the polarization time without reducing the polarization efficiency, we established the dynamic equation of Larmor motion of hydrogen proton magnetization and attempted to obtain the optimized polarization pulse waveform through numerical calculation of the equation, thereby achieving fast measurement. For this purpose, we introduced the polarization saturation factor *θ* to characterize the degree of proton magnetic moment alignment, which enabled us to optimize the 90° DC pulse parameters to obtain the best polarization efficiency. The calculated results show that the hydrogen proton magnetic moment alignment can be rapidly completed by optimized polarization pulse waveform, pulse strength, and pulse duration. The waveform index *α*, pulse strength *B*_0_ and pulse duration *t*_p_ after theoretical optimization is 3, 3 mT, and 3 ms, respectively. Optimized parameters are used in JOM-5SF OVM, and a continuous and stable Larmor signal with an SNR of 44 is obtained. Experimental sensitivity estimation in nature and artificial magnetic field show that the sensitivity is 0.021 nT@1Hz by using the proposed 90° DC pulse waveform.

Both theoretical and experimental results indicate that polarization time is closely related to the dynamic process of molecular magnetization. By using optimized 90° pulse based on the theory given in this paper, proton magnetic moment alignment can be rapidly completed on the order of milliseconds, significantly reducing polarization time for fast measurement.

Furthermore, the proposed dynamic equation analysis method for hydrogen protons is not limited to the Overhauser magnetometer; it is fundamentally applicable to other nuclear precession magnetometers based on similar physical principles.

## Figures and Tables

**Table 1:** Optimized parameters of 90° pulse.

Parameter	Recommended Ranges	Recommended Values
Waveform index α	30 > *α* > 2	*α* = 3
Pulse strength *B*_0_	*B*_0_ ≥ 3 mT	*B*_0_ = 3 mT
Pulse duration *t*_p_	*t*_p_ ≥ 3 ms	*t*_p_ = 3 ms

**Table 2:** Error evaluation of the proposed JOM-5SF Overhauser magnetometer.

Standard Magnetic Field	JOM-5SF Average	Absolute Error
20,577.53 nT	20,577.51 nT	−0.02 nT
40,498.31 nT	40,498.34 nT	0.03 nT
50,180.61 nT	50,180.51 nT	−0.1 nT
61,407.26 nT	61,407.21 nT	−0.05 nT
81,949.21 nT	81,949.23 nT	0.02 nT

## Data Availability

No new data were created or analyzed in this study. Data sharing is inapplicable to this article.
